# Biodegradable Polymers and Stem Cells for Bioprinting

**DOI:** 10.3390/molecules21050539

**Published:** 2016-04-29

**Authors:** Meijuan Lei, Xiaohong Wang

**Affiliations:** 1 Center of Organ Manufacturing, Department of Mechanical Engineering, Tsinghua University, Beijing 100084, China; lmj15@mails.tsinghua.edu.cn; 2 Center of 3D printing & Organ Manufacturing, Department of Tissue Engineering, China Medical University (CMU), Shenyang 110122, China

**Keywords:** bioprinting, organ manufacturing, stem cells, gelatin based hydrogels, tissue engineering

## Abstract

It is imperative to develop organ manufacturing technologies based on the high organ failure mortality and serious donor shortage problems. As an emerging and promising technology, bioprinting has attracted more and more attention with its super precision, easy reproduction, fast manipulation and advantages in many hot research areas, such as tissue engineering, organ manufacturing, and drug screening. Basically, bioprinting technology consists of inkjet bioprinting, laser-based bioprinting and extrusion-based bioprinting techniques. Biodegradable polymers and stem cells are common printing inks. In the printed constructs, biodegradable polymers are usually used as support scaffolds, while stem cells can be engaged to differentiate into different cell/tissue types. The integration of biodegradable polymers and stem cells with the bioprinting techniques has provided huge opportunities for modern science and technologies, including tissue repair, organ transplantation and energy metabolism.

## 1. Introduction

On the basis of statistical data, there were 25.7 million adults and 75,000 children suffering from cancer of stomach, liver, breast, lung, and so on during 1995–2009 [1]. There were 117,040 patients that needed organ transplantation, but only 28,053 suitable organs available in the USA in 2013 [2]. Besides huge economic costs and immunological rejections of allografts, organ shortage has become a great restriction in organ transplantation. For the shortcomings and limitations of the surgical transplantation, scientists have tried various new methods to increase man’s life span. These new methods include biomaterial induction, cell therapy, tissue engineering and organ manufacturing approaches [3,4,5,6].

More and more evidence has shown that stem cells are the building blocks or backbones for regenerative medicine with their unique functions and advantages of self-renewal, high proliferation, and multiple-directional differentiation abilities [7,8]. The combination of biodegradable polymers with multinozzle 3D bioprinting technologies (*i.e.*, cell assembling techniques) gives the hope to deposit various cells in place to mimic normal biological conditions *in vitro* and *in vivo* [9,10,11,12,13,14,15,16].

Recently, complex tissue constructs, such as vascularized liver, adipose, bone, cartilage and muscle tissues, have been fabricated by an integrated tissue-organ printer (ITOP) [17,18]. Bioprinting technology has been regarded as the most effective tool for regenerative medicine including tissue engineering and organ manufacturing. Some scientists have considered bioprinting technology as the conjunction of 3D printing and organ manufacturing techniques [19,20,21,22].

The main characteristic of bioprinting technology is printing cells and extracellular matrices (ECMs) layer by layer to form 3D tissue/organ-like constructs. Cells in bioprinting can be adult cells or stem cells extracted from the patient who requires organ transplantation, which solves the rejection problems that arise from the recipient's immune system. Excelling at tissue engineering, bioprinting can create customized structures with the computer-aided models (CAD) quickly, and print cells, cytokines, or ECMs automatically and precisely. Hence, this technology has been regarded as a forward-looking method to assemble cells and biomaterials rapidly and precisely [19,20,21,22].

However, organ manufacturing for organ replacement and reconstruction has not yet been completely applied to clinical treatment with reliable bioartificial organs [23,24]. Especially, functional branched vascular system manufacturing remains a technological barrier that needs to be broken through immediately. At present, most of the existing technologies are limited in simple tissue manufacturing and high-throughput drug screening areas [9,10,11,12,13,14,15,16,25]. Furthermore, 3D bioprinting involves in various aspects such as cell types, biomaterials, and growth factors, of which each factor plays a key role in determining the printing results. The selection of biomaterials is of fundamental importance in bioprinting, which contains a selection of adult or stem cells, synthetic or natural polymers, and growth factors.

3D bioprinting approaches include mechanical enhancement, biomimicry, autonomous self-assembly and mini-tissue formation stages. It brings hope to humanity of biomimetic structures with specially designed patterns, material composition and degradation kinetics, controllable mechanical properties and biological effects. Most importantly, it is possible to obtain complex tissue/organ structures with physical and chemical properties similar to their counterparts with the combination of various factors for tissue and organ regeneration [9,10,11,12,13,14,15,16]. Thus, there are four basic elements in bioprinting technologies: cells, growth factors, biodegradable polymers and bio-printer. Different organ manufacturing technologies differ in these four aspects. Generally, cells, as “bio-inks”, are mixed in biodegradable polymer hydrogels before being printed [9,10,11,12,13,14,15,16].

## 2. Classification of Bioprinting Techniques

**Figure 1:**
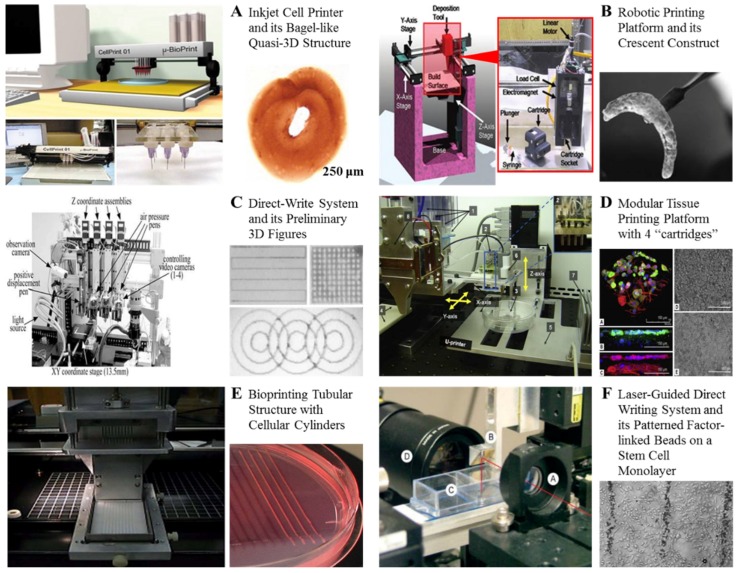
(**A**) A inkjet cell printer and its bagel-like quasi-3D structure [9]; (**B**) A robotic printing platform and its crescent construct [9]; (**C**) A direct-write system and its preliminary 3D figures [9]; (**D**) A modular tissue printing platform with four “cartridges” to load cell suspensions or hydrogels developed in Brigham and Women’s Hospital, Harvard Medical School, Prof. Yoo’s group [9]; (**E**) A bioprinting tubular structure with cellular cylinders developed in University of Missouri, Columbia, USA, Prof. Forgacs’ group [9]; (**F**) A laser-guided direct writing (LGDW) system and its patterned factor-linked beads on a stem cell monolayer with micrometer accuracy (Bar = 200 μm) developed in University of Minnesota, Prof. Odde’s group [9].

Based on the working principles, bioprinting technologies have been divided into four classes: inkjet bioprinting, extrusion bioprinting, laser-assisted bioprinting, and ultrasonic bioprinting (Figure 1) [9,10,11,12,13,14,15,16]. Among them, the former three are commonly employed in modern tissue engineering and organ manufacturing areas. In detail, inkjet bioprinting technology is based on simple home printing techniques. Cells and biomaterials such as hydrogels are printed separately layer by layer to form an object using thermal or acoustic methods [26]. Tissues/Organs can be gradually matured when the cells communicate and connect to each other after printing (Figure 1A). During thermal inkjet printing, heat is generated at the printer head and the cells and biomaterials are forced out of the nozzle through pressure pulses. The system temperature can rise 4–10 °C with no obvious detrimental effect on cell viability [27,28].

In laser-assisted bioprinting or laser bioprinting, a bubble between a solution and a piece of glass is usually created through a vapor pressure (*i.e.*, laser pulse). This pressure shoots a small drop of the solution, including cells, towards the collector substrate. Drop by drop, a tissue-like structure can be produced through the repeated processes. Laser bioprinting has the advantage of high resolution in living cell arrangement. In the meantime, cells undergo thermal and mechanical deformation from the laser during the printing processes. Usually, the damage can be decreased to some degree through incorporating biodegradable polymers in the “bio-inks”. Especially, with scaffold biomaterials employed, elegant 3D structures can easily be printed [29,30]. Laser bioprinting can also be employed for multiple cell printing using different cell types in the substrate, which is a vital element for tissue engineering and organ manufacturing (Figure 1F).

Extrusion-based bioprinting is obviously based on the extrusion principle. Fluids, released by a pressure-assisted system, are extruded automatically via a three-axis robotic system to an intended position. There exist multinozzle-based bioprinting systems, which are identified as the forward-looking methods to assembly cells and extracellular matrices (ECMs) precisely (Figure 1D) [9,10,11,12,13,14,15,16,31,32,33,34,35].

In general, different bioprinting techniques have dissimilar printing themes and limitations [31,32,33,34,35]. For instance, diversity and affordability are the prominent features of an inkjet bioprinting technique. Different cells and ECMs can be deposited together or separately. When cells are printed *in situ* (e.g., in patient body), there is no need to have a flat surface or platform to support the outcomes [33]. However, only liquids with low viscosities and low cell numbers can be printed to avoid clogging in the nozzle and to reduce shear stress on the cells. In extrusion bioprinting, the structural integrity of cells and ECMs obviously precedes those in inkjet bioprinting. Multiple cell types with high cell densities can be printed simultaneously using multinozzle printers. It is possible to directly print organs with a branched vascular system because of cells encapsulated in the intrinsic connectible ECMs or scaffold materials [34,35]. Nevertheless, the limited ECM selection and short storage time period of biodegradable polymer hydrogels have an impact on the technical development and practice [9,10,11,12,13,14,15,16,31,32,33,34,35,36]. In laser-assisted bioprinting, a broad range of biomaterials with various viscosities can be printed without clogging problems. However, the cell printing efficiency needs to be improved [37].

Currently, some achievements have been attained with various cells and biodegradable polymers. For example, adipose-derived stem cells (ADSCs) and mesenchymal stem cells (MSCs) were printed using a laser-assisted bioprinting (LaBP) technique [38,39]. Around five to seven living cells were printed using a laser-induced forward transfer technique with high precision at micron scales [40]. Human microvascular endothelial cells together with fibrin were printed using a thermal inkjet printer [41]. An aortic valve-like structure was printed using a microextrusion bioprinter [42]. In parallel, the integration or combination of different cells and biodegradable polymers for extrusion-based 3D bioprinting techniques has been extensively exploited by our group [43,44,45,46,47,48,49,50,51,52,53,54,55,56,57,58].

## 3. Biodegradable Polymers in Bioprinting

Biodegradable polymers can be divided into synthetic polymers (such as polyurethane (PU) and poly(lactic-co-glycolic acid) (PLGA)), and natural polymers (such as gelatin and collagen). Both have been widely used in bioprinting techniques with profound influence in cellular activities, histogenese modulation and tissue/organ generation [43,44,45,46,47,48,49,50,51,52,53,54,55,56,57,58]. A distinction between the two polymers is that natural polymers are born with bioactivities; on the contrary, synthetic polymers are generally inert. As a result, synthetic polymers are superior to natural polymers in terms of immunogenic responses and mechanical strengths.

The commonly used biodegradable polymers in bioprinting are gelatin, collagen, alginate, chitosan, fibrin, hyaluronic acid and their mixtures, which can be changed from sol to gel states in response to various external stimuli, such as temperature, light, pH, magnetism and electricity [59,60,61]. As cell loading ECMs or accommodations, these polymers possess the essential characteristics, such as bioactive; biocompatibility; controllable biodegradability (hydrolytically or enzymatically degradable); nontoxic; stable; processibility; storability, and suitable physical (*i.e.*, mechanical, structural, and topological), chemical (*i.e.*, crosslinking) and biological properties [9,10,11,12,13,14,15,16,31,32,33,34,35,36,43,44,45,46,47,48,49,50,51,52,53,54,55,56,57,58,62,63,64,65]. Some other biodegradable polymers, such as dextran, starch, resin, agar, matrigel, pluronic F-127, polypeptide-DNA, and poly(acrylic acid), have also been used in bioprinting [66,67,68,69]. Besides providing cells with necessary nutrients, water and oxygen, the polymers also play an important role in supporting the printed structures.

Most of the natural biodegradable polymers can be dissolved in cell friendly inorganic solvents to form solutions or hydrogels with good biocompatibility and biodegradability. The solution or hydrogel states of the biodegradable polymers have certain fluidity during bioprinting [43,44,45,46,47,48,49,50,51,52,53,54,55,56,57,58]. The mobility of the polymer solutions or hydrogels can be modified in rapid prototyping (RP) devices, and can be manipulated with high resolution and mature accuracy. This is vital important for the biological property retention and tissue/organ recreation during and after cell printing processes [70,71]. These polymers are mainly applied to temporary prosthesis, scaffold formation and drug delivery fields. Several relevant parameters of biodegradable polymers, such as extreme temperatures, organic solvents, and water deficiencies can negatively influence cell viabilities.

Usually, different biodegradable polymers have different printing properties. Certainly, each bioprinting machine has its own taste of biomaterials to match. To some extent, the processibility of the biodegradable polymers determines the availability of biomaterials. Hydrogel, a water-swollen, and cross-linked polymeric network produced by the simple reaction of one or more monomers [9,10,11,12,13,14,15,16,31,32,33,34,35,36,43,44,45,46,47,48,49,50,51,52,53,54,55,56,57,58,62,63,64,65,72], plays increasingly role in organ manufacturing as well as bioprinting technologies. In our experience, various hydrogels, typically biodegradable natural or synthetic polymeric solutions, are ideal choices for scaffold biomaterials, which are highly hydrated (water content ≥ 30% by weight) and easily manipulated [9,10,11,12,13,14,15,16,31,32,33,34,35,36,43,44,45,46,47,48,49,50,51,52,53,54,55,56,57,58,62,63,64,65,73]. For example, gelatin, with properties such as biocompatible, biodegradable, solubility in water, and a melting point at about 28 °C, is very flexible but strong in hydrogel states, which can easily be manipulated and modified, preventing large molecules diffusion inside randomly [74,75]. As an alternative, collagen is also a fascinating biomaterial, which is rich in mammalian cells and ECM components [76,77]. For its low toxicity and fast-acting property, alginate has been used frequently for cell encapsulation and drug delivery [43,44,45,46,47,48,49,50,51,52,53,54,55,56,57,58,78]. To date, the exploration of new biomaterials for tissue and organ printing is still underway [52,53,54,55,56,57,58].

Emphasis should be given to those ECMs that play a significant role in cell survival, proliferation, migration and differentiation during and after bioprinting processes, with the principal means of mechanical support and biochemical signals [79,80]. Many biodegradable polymers combinations, mimicking the properties of ECMs, have been designed for different cell printing [81,82,83]. In detail, some ECM-like hydrogels have been widely used in the research areas, such as drug delivery, tissue engineering and organ manufacturing, to promote nutrient diffusion, cell migration, angiogenesis, wound healing, and tissue/organ regeneration [5,6,7,8,9,10,11,12,13,14,15,16,17,18,19,20,21,22,23,24,43,44,45,46,47,48,49,50,51,52,53,54,55,56,57,58,84].

Normally, the printing temperatures of cells encapsulated in the ECM-like polymer hydrogels should be between 1 °C and 37 °C, in order to avoid ice nucleation or overheat harms to cells [5,6,7,8,9,10,11,12,13,14,15,16,17,18,19,20,21,22,23,24,43,44,45,46,47,48,49,50,51,52,53,54,55,56,57,58,85]. *In vivo* stabilities, compatibilities and degradation rates of polymer hydrogels should be seriously considered before the 3D constructs can be implanted, particularly for soft tissues or organs, such as the liver, heart, and pancreas. Sometimes, the moisture content and permeability of the polymer hydrogels also need to be considered.

For example, alginate, also named as algin or alginic acid, is an anionicpolysaccharide that has been widely used as an ion-sensitive hydrogel for cell encapsulation in drug delivery and bioprinting [86,87,88]. A remarkable feature of alginate is its ability to be rapidly crosslinked in a cell-endured condition upon contact with divalent cations, such as calcium, strontium, and barium and zinc ions. However, the crosslinking processes are reversible. When the resulted complexes are cultured in liquid media and the chelated divalent cations are released from the hydrogels, the printed structures cannot be maintained for a long period. To solve this problem, we have further incorporated crosslinkable chitosan or polymerizable fibrinogen in the gelatin based cell-printing matrices [45,46,47,48,49,50,51,52,53,54,55,56,57,58]. Besides being ion-crosslinked, the alginate-based hydrogels can also be photo-crosslinked or enzyme-crosslinked [45,46,47,48,49,50,51,52,53,54,55,56,57,58,88,89]. In addition, to increase the biocompatibilities of alginate, some researchers have modified alginate hydrogels with cell-adhesive peptide, such as RGD, or ECM-like components, such as gelatin or collagen [90].

In our group, we have chosen gelatin based natural biodegradable polymers, such as gelatin/chitosan, gelatin/alginate, gelatin/hyaluronan, gelatin/alginate/chitosan and gelatin/alginate/fibrinogen as the main component of ECM for various extrusion-based cell-printing techniques [5,6,7,8,9,10,11,12,13,14,15,16,17,18,19,20,21,22,23,24,43,44,45,46,47,48,49,50,51,52,53,54,55,56,57,58]. Tailor-made nozzles or thin syringe needles have been employed to print the cell-laden hydrogels. The printing processes have nearly no harm to the encapsulated cells. Glycerol, dextran, dimethyl sulfoxide and other bioactive factors can be incorporated into the gelatin-based hydrogels directly [43,44,45,46,47,48,49,50,51,52,53,54,55,56,57,58,85]. Gelatin is a denatured, biodegradable polypeptide derived from partial hydrolyzed collagen, which is widely found in human tissues [9,10,11,12,13,14,15,16,31,32,33,34,35,36,43,44,45,46,47,48,49,50,51,52,53,54,55,56,57,58,62,63,64,65,72]. The special thermoresponsive property of gelatin allows the hydrogels to be printed layer by layer in a controlled manner. All cell types can be encapsulated in the gelatin based sol-gel hydrogels for complex organ construction. Branched vascular systems can be obtained with a synthetic polymer overcoat for anti suture purpose [22,23,24,56,57]. Nevertheless, the gel states of the gelatin-based hydrogels are not stable when the environmental temperature is over 20 °C or the printed constructs are put into a culture medium. Consequently, the cell-laden hydrogels need to be crosslinked to yield a defined shape or a stable 3D structure after printing [18,43,44,45,46,47,48,49,50,51,52,53,54,55,56,57,58,59].

**Figure 2:**
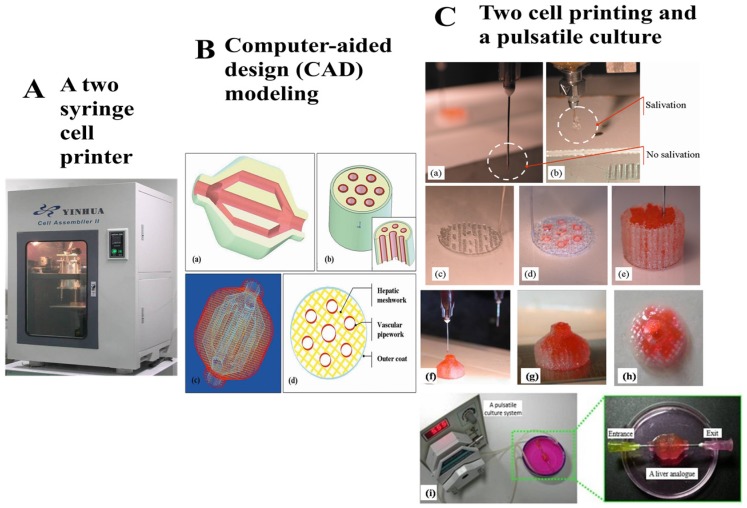
A two-nozzle 3D bioprinting technique developed in Tsinghua University Prof. Wang’s group. Two different cell types in the gelatin-based hydrogels have been assembled simultaneously into a construct [10]. (**A**) The two-syringe/nozzle cell printing machine; (**B**) A computer-aided design model; (**C**) Adipose-derive stem cells and hepatocyte have been printed together with a branched vascular system and pulsatile cultured after printing.

**Figure 3:**
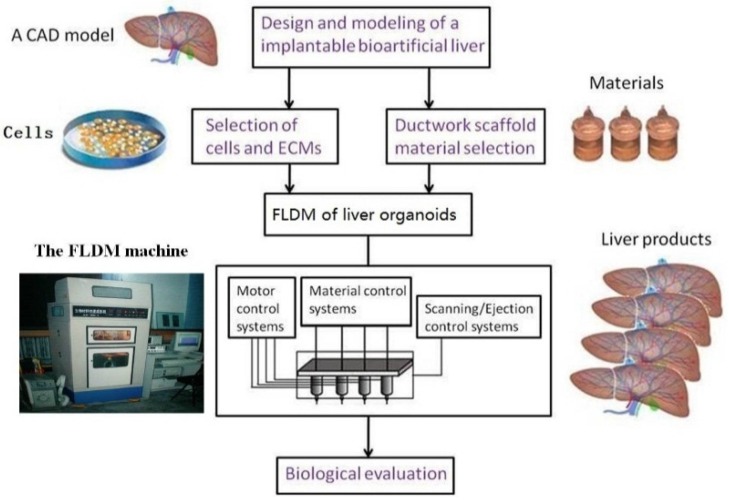
Schematic description of the modeling and manufacturing processes of liver lobes with a four-nozzle low-temperature deposition manufacturing (FLDM) system developed in Tsinghua University, Prof. Wang’s group [10,11].

Evidently, the number of biomaterials that can be simultaneously printed depends mainly on the nozzle numbers of an extrusion-based bioprinter. Different material systems can be integrated into a construct by choosing proper printing parameters. For example, in our group, we have developed various multinozzle bioprinters for different organ manufacturing. Especially, two robotic arm controlled injectable nozzles and two extrusion-based syringes have been combined for solid organs, such as the breast, liver and heart, manufacturing [91]. We have assembled various cells, such as ADSCs and hepatocytes, using our homemade multiple nozzle cell 3D printers, to produce complex organ precursors with branching vascular systems inside, and to convert ADSCs into vascular endothelial cells and smooth muscle cells in the 3D constructs [22,23,24]. Figure 2 is an illustration of a two-syringe RP technique [10,48]. Figure 3 is an illustration of liver manufacturing with a four-nozzle bioprinter and multiple biomaterials. Four different biomaterials can be simultaneously printed together.Thereby, it can meet the requirements for stable vascular system formation within a hybrid hierarchical polyurethane-cell/hydrogel construct [10,11]. Correspondingly, it was the first time some new words, such as “inner scaffold” and “outer scaffold”, have appeared in our manuscripts and cover letters. With the similar integrations of extrusion nozzles and biomaterials, Kang and coworkers have produced human scale bone, cartilage and muscle tissues with a composite gelatin/fibrinogen/hyaluronan/glycerol hydrogel [17]. The combination of different biodegradable polymers in one hydrogel has been proved to be an effective way in 3D bioprinting techniques, especially for complex organ manufacturing.

## 4. Stem Cells in Bioprinting

There is a current trend to combine stem cells and growth factors with ECMs in 3D milieus to mimic organ development [92,93,94,95,96,97,98,99,100,101,102,103,104,105]. In fact, with the proper use of growth factors, stem cells can be induced into multiple tissues with a cocktail growth factor engagement approach [22,23,24,52,53,54,55,56,57,58]. With this approach, stem cells are regarded as the ideal cell types for bioprinting.

Especially, embryonic stem cells (ESCs) have been used for developmental biology, regenerative medicine, cell replacement therapy and drug discovery with high differentiation potentialities [106]. However, in view of the moral issue, the applications of ESCs in 3D bioprinting techniques have been greatly influenced. Currently, some somatic stem cells, such as adipose-derived stem cells (ADSCs) and induced pluripotent stem cells (iPSCs), are becoming increasingly popular and attractive [107,108,109,110,111,112].

ADSCs are easily accessible from mammalian epithelial tissues in large quantities [113,114,115,116,117,118]. ADSCs can differentiate into cardiac cells/ muscles, endothelial cells/tissues, smooth cells/muscle, and be used in many bioapplications. In addition, ADSCs have extra lineage differentiations, including adipocytes, chondrocytes, hepatocytes, pancreatic cells, neuronal-like cells, osteoblasts, pancreas cell lines and bone-related cells [119,120,121]. As stated above, in our group, we have incorporated ADSCs in various natural biodegradable polymer hydrogels for complex organ manufacturing [9,10,11,12,13,14,15,16,48,49,50,51,52,53,54,55,56,57,58]. Extremely high cell viabilities can be achieved by optimizing the printing parameters. The printing processes have no adverse effects on the stem cell proliferation and differentiation abilities. Multiple tissues can be easily coordinated in the printed construct due to the growth factor incorporation before printing or cocktail growth factor engagement after printing [9,10,11,12,13,14,15,16,48,49,50,51,52,53,54,55,56,57,58]. Particularly, the location or spatial effects of growth factor engagement are prominent for the printed stem cells [6,22,23,24]. All the stem cells can be engaged to differentiate into target cell types in the constructs. Different vascular segments can be printed with different extrusion nozzles and biomaterials. It is therefore the most effective way for complex organ printing [6,7,8,9,10,11,12,13,14,15,16,22,23,24].

iPSC is a perfect cell type for its feature of overcoming the difficulty of the limitations associated with the current cell sources and has developed very quickly. iPSCs have the potential to differentiate into almost all kinds of cells for different tissues, such as nerve, blood, bone, and heart, under appropriate conditions. Currently, iPSC lines of rats [122], pigs [123], humans [124], monkeys [125] and rabbits [126] have been set up successfully. Some researchers have chosen to focus on iPSCs for disease model establishment, drug discovery template, energy metabolic research, cell therapy and organ manufacturing. In detail, the methods of generating integration-free iPSCs are as followings: non-integrating DNA methods, RNA-based methods, proteins-based methods [127,128,129]. Similar to other cell printing techniques, 3D stem cell printing can provide stem cells with complex 3D cellular microenvironments similar to their native counterparts. Patient self-derived stem cells can solve the immune rejection issues.

Thus far, the research about stem cells and regenerative medicine is increasingly piling up, which brings more opportunities and injects new vitalities to effectively treat end failure organs [6,7,8,9,10,11,12,13,14,15,16,18,19,20,21,22,23,24,25,26,27,28,29,30,31,32,33,34,35,36,37,38,39,40,41,42,43,44,45,46,47,48,49,50,51,52,53,54,55,56,57,58,98,99,100,101,102,103,104,105,106]. Consequently, the number of published papers and financial support from government have increased dramatically [130].

## 5. Future Outlook

Bioprinting technology has obtained growing popularity for its favorable meanings. Some groups have made great progress with the pertinent techniques in tissue engineering, organ manufacturing and drug screening areas. Biodegradable polymers are usually used as the cell printing ECMs or accommodations, while stem cell techniques are especially helpful in complex organ manufacturing. The development and future of bioprinting techniques are promising: (1) it can solve the donor shortage problems of organ transplantation; (2) it can overcome the immune rejection symptoms caused by allograft tissue/organ transplantation; (3) it can reduce the high cost and heavy burden of the patients with reverse engineering and personal manufacturing techniques; (4) it can establish a platform for high-throughput drug screening and organ manufacturing; and (5) it can be easily accepted by researchers and doctors with those CAD tissue/organ models.

Nevertheless, the development of bioprinting is still imperative and challenging. In particular, several aspects should be further addressed: (1) lack of design principles for bioprinting despite an emergency in additive manufacturing areas [131,132,133]; (2) further 4D, 5D even 6D development based on the 2D and 3D printing techniques [134,135]; (3) optimal stem cells induction approaches before, during or after the printing processes; (4) multiple cell type or biomaterial system incorporation protocols for complex tissue/organ manufacturing; (5) special cell culture incubators to acquire large amount of cells in a short time period; (6) biological functionality realization within the 3D printed substitutions; (7) harsh environment factor reduction (such as temperature and pressure) for biological property maintenance of the integrated cells and growth factors; and (8) typical zone or layer designs for complex organ products. Further multidisciplinary research and cooperation are still urgently needed relating to biology, chemistry, medicine, materials, mechanics, and pathophysiology sciences and technologies. More and more high-throughput manufacturing products, such as the liver, heart, kidney, skin, cartilage and bones, will become possible with promising 3D bioprinting technologies. We are herein expecting a bright and fruitful future for the modern multinozzle 3D bioprinting techniques.
